# Allele-specific quantification of human leukocyte antigen transcript isoforms by nanopore sequencing

**DOI:** 10.3389/fimmu.2023.1199618

**Published:** 2023-08-18

**Authors:** Andrew E. O. Hughes, Maureen C. Montgomery, Chang Liu, Eric T. Weimer

**Affiliations:** ^1^ Department of Pathology and Immunology, Washington University School of Medicine, St. Louis, MO, United States; ^2^ Molecular Immunology Laboratory, McLendon Clinical Laboratories, University of North Carolina Hospitals, Chapel Hill, NC, United States; ^3^ Department of Pathology and Laboratory Medicine, University of North Carolina at Chapel Hill School of Medicine, Chapel Hill, NC, United States

**Keywords:** human leukocyte antigen (HLA), nanopore sequencing, long-read sequencing, transcript isoforms, allele-specific expression

## Abstract

**Introduction:**

While tens of thousands of HLA alleles have been identified by DNA sequencing, the contribution of alternative splicing to HLA diversity is not well characterized. In this study, we sought to determine if long-read sequencing could be used to accurately quantify allele-specific HLA transcripts in primary human lymphocytes.

**Methods:**

cDNA libraries were prepared from peripheral blood lymphocytes from 12 donors and sequenced by nanopore long-read sequencing. HLA reads were aligned to donor-specific reference sequences based on the known type of each donor. Allele-specific exon utilization was calculated as the proportion of reads aligning to each allele containing known exons, and transcript isoforms were quantified based on patterns of exon utilization within individual reads.

**Results:**

Splice variants were rare among class I HLA genes (median exon retention rate 99%–100%), except for several *HLA-C* alleles with exon 5 spliced out of up to 15% of reads. Splice variants were also rare among class II HLA genes (median exon retention rate 98%–100%), except for *HLA-DQB1*. Consistent with previous work, exon 5 of *HLA-DQB1* was spliced out in alleles with a mutated splice acceptor site at rs28688207. Surprisingly, a 28% loss of exon 5 was also observed in *HLA-DQB1* alleles with an intact splice acceptor site at rs28688207.

**Discussion:**

We describe a simple bioinformatic workflow to quantify allele-specific expression of HLA transcript isoforms. Further studies are warranted to characterize the repertoire of HLA transcripts expressed in different cell types and tissues across diverse populations.

## Introduction

Human Leukocyte antigens (HLA) are encoded by the most polymorphic group of genes in the human genome. The genetic diversity of these molecules enables the presentation of a broad spectrum of peptides to launch adaptive immunity. HLA molecules are also the main immunological barrier to allogeneic transplantations due to their wide tissue expression and strong immunogenicity. Therefore, a thorough genetic characterization of HLA genes is essential for understanding the immune response in healthy and diseased states and in the transplant setting. While extensive efforts have been made to catalog the large and growing number of HLA alleles in the human population, our understanding of the functional aspect of HLA genetic diversity is limited (1). The expression and function of HLA molecules could be affected by their epigenetic modifications, transcriptional and post-transcriptional regulations, differential splicing, and post-translational modifications, which may be explored to reveal novel mechanisms of diseases. It has been observed that all human multiexon genes show alternative splicing, and spliced genes have over nine alternative transcripts on average (2). This proof-of-concept study focuses on characterizing the alternative splicing of HLA genes in primary human lymphocytes.

Alternative splicing has been traditionally examined by short-read RNA sequencing (RNA-seq) on the Illumina platform due to its high read-level accuracy, cost-effectiveness, and availability of bioinformatics tools for data analysis. However, the typical length of Illumina read ranges from 150-300 bp, making it challenging to detect exon-exon junctions or resolve complex alternative splicing events. The limited dynamic range of Illumina sequencing in this setting reduces the sensitivity of detecting low-abundance splicing isoforms, necessitating ultra-deep sequencing to achieve adequate power for splicing analysis at the genome scale (3). The bioinformatic pipelines for splicing analysis may also introduce artifacts and generate false positive results (4). These limitations may be aggravated when studying the alternative splicing of diverse HLA alleles with densely packed sequence variants and variable expression levels.

The advent of long-read sequencing technologies, such as Pacific Biosciences’ single-molecule real-time (SMRT) sequencing and Oxford Nanopore Technologies’ nanopore sequencing, may drastically improve the sensitivity and specificity of splicing analysis by detecting full-length isoforms. These platforms can routinely produce sequence reads of tens of thousands of bases, which is ideal for full-length sequencing of entire transcripts. Nanopore sequencing has been limited by a lower raw read accuracy, with a median of 87.7% and 93.7% on the R9.4 and R10.3 flow cells, respectively (5). However, nanopore sequencing has the potential to deliver rapid, point-of-care sequencing without batching (6). We have previously demonstrated the feasibility of generating accurate high-resolution HLA typing and quantifying HLA allelic expressions by nanopore sequencing (5, 7–9). Here, we report a novel and straightforward workflow to survey the exon utilization across diverse alleles at 11 classical HLA loci and quantify the relative abundance of phased transcript isoforms by nanopore-based RNA-seq of primary human lymphocytes.

## Materials and methods

### RNA-seq of human lymphocytes

Studies involving human participants were reviewed and approved by the Institutional Review Board and Office of Human Research Ethics at the University of North Carolina at Chapel Hill (IRB #18-0691).

RNA-seq was performed on primary human lymphocytes as described previously (8). Briefly, total lymphocytes were isolated from peripheral blood from 12 healthy donors using the EasySep Direct Human Total Lymphocyte isolation kit (StemCell Technologies, Vancouver, BC, Canada). In addition, a second (replicate) sample was collected from three donors to evaluate reproducibility. mRNA was extracted from up to 1E7 cells using the GenElute Direct mRNA MiniPrep Kit (Sigma-Aldrich, St. Louis, MO) according to manufacturer instructions. cDNA synthesis and sequencing library preparation were performed using the SQK-PCS108 cDNA-PCR Sequencing Kit (Oxford Nanopore Technologies, Oxford, UK). Libraries were sequenced on MinION FLO-MIN106D R9.4.1 flow cells (Oxford Nanopore Technologies, Oxford, UK) using MinKNOW (v3.1.13) for an average of 18 hours (range 16–22 hours). Up to two samples were sequenced per flow cell. Base calling was performed with Albacore (v2.3.4) or Guppy (v2.3.1). Adapter sequences were trimmed with Porechop (v0.2.3).

### Reference HLA typing

HLA types for both class I and class II loci were determined by short-read sequencing from genomic DNA using AlloSeq Tx17 (CareDx, San Francisco, CA) for library preparation followed by sequencing on a MiSeq with 2x150 base pair reads (Illumina, San Diego, CA) (10).

### Allele-specific quantification of HLA transcript isoforms

For each sample, RNA-seq reads were first aligned to all nucleotide coding sequences from the IPD-IMGT/HLA database (v3.41) using minimap2 (with the options -x splice and –secondary=no) to identify candidate HLA reads. Reads aligning to any HLA transcript were then realigned to sample-specific genomic DNA references based on known HLA types (with the options -x splice, –secondary=no, and providing the location of known splice junctions *via* –junc-bed). For each allele, the IPD-IMGT/HLA allele with the lowest four-field nomenclature consistent with the known type was used as the reference sequence. Genomic DNA sequences for all alleles in the study cohort were available in the IPD-IMGT/HLA database.

For each reference allele, the proportion of reads containing known exons was calculated by intersecting the coordinates of known exons with the coordinates of aligned reads using bedtools (v2.29.2) (11). For each read, exons were counted as retained in a transcript if they overlapped any portion of the aligned sequence. In addition, exons were counted as excluded from a transcript only if the aligned sequence overlapped at least one exon 5’ and at least one exon 3’ of the exon of interest (i.e., 5’ or 3’ truncation was not considered evidence of splicing). Only alleles with ≥20 reads (class I) or ≥10 reads (class II) were included in this analysis. For transcript quantification, only reads spanning all known exons were included (i.e., reads with 5’ or 3’ truncation were omitted).

### Data visualization and statistical analysis

Statistical analysis was performed in R (v4.1.0) using base packages as well as the tidyverse package (v1.3.1). Sashimi plots were generated using ggsashimi (v1.1.5) (12). Histograms, scatter plots, and tables were prepared in R (v4.1.0) using the packages ggplot2 (v3.3.6), knitr (v1.39), and kableExtra (v1.3.4).

## Results

**Figure 1:**
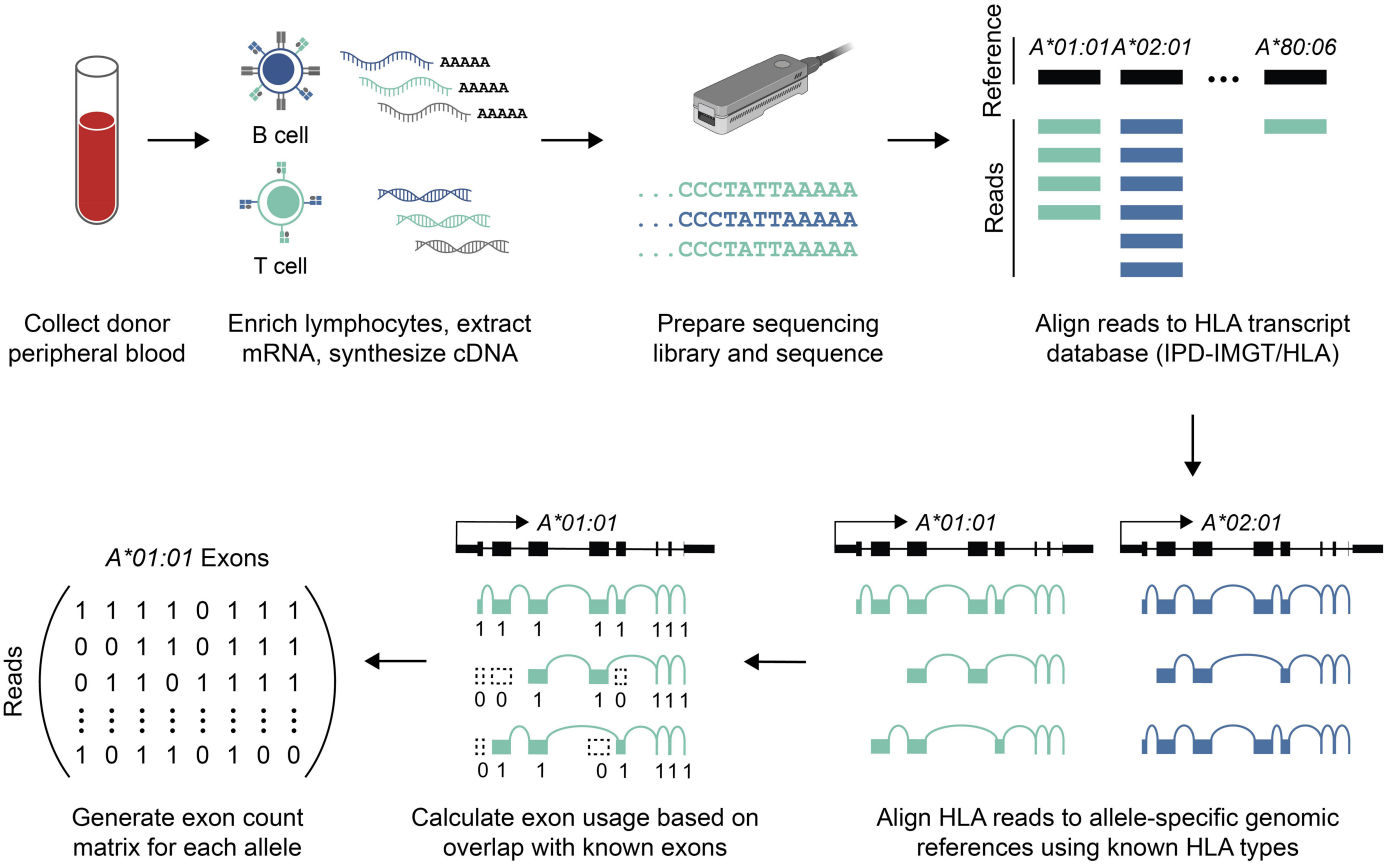
Data generation and bioinformatic analysis. Schematic overview of sequencing library preparation and data analysis (see **Methods**). Briefly, mRNA was isolated from peripheral blood lymphocytes, and the full-length cDNA library was sequenced using nanopore long-read sequencing. Reads aligning to HLA genes were identified, and those reads were re-aligned to patient-specific references to estimate allele-specific coverage. Allele-specific alignments were then used to calculate exon usage.

To characterize the landscape of HLA transcripts in healthy adults, we analyzed previously generated long-read RNA-seq data from peripheral blood lymphocytes (**Figure 1**) (8). This dataset included RNA-seq from 12 independent donors as well as replicate samples from three donors. HLA types for all samples were determined by short-read sequencing of genomic DNA for both class I and class II HLA loci (**Supplemental Table 1**). To estimate the rate of allele-specific exon usage for both class I and II HLA genes for each donor, RNA-seq reads were first aligned to all nucleotide coding sequences from the IPD-IMGT/HLA database (1). Candidate HLA reads were then realigned to sample-specific references comprising only the genomic DNA sequences of known donor alleles. Finally, the proportion of reads containing known exons was calculated for each allele (see **Materials and Methods**).

**Figure 2:**
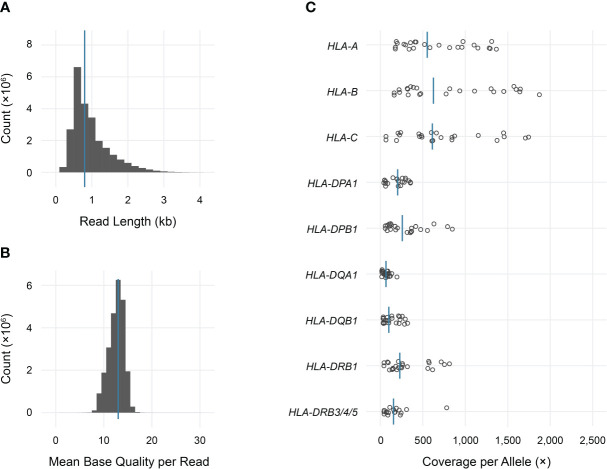
Sequencing data characteristics and HLA gene coverage. **(A)** Read length histogram (median 0.8 kb). **(B)** Mean base quality histogram (median 14). **(C)** Dot plot of total coverage per allele for each sample in the study. Bars indicate medians.

**Figure 3:**
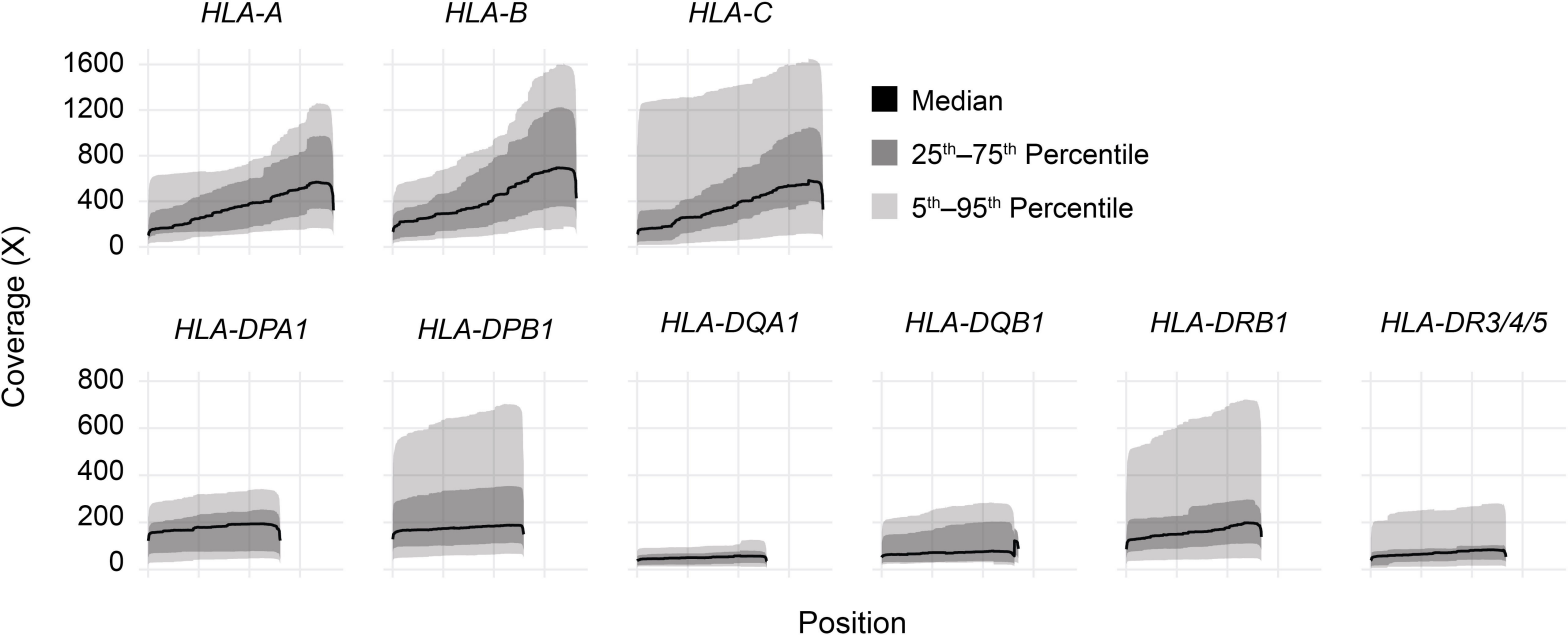
HLA gene body coverage shows 3’ bias. Smoothed coverage by position for all HLA genes analyzed in this study. Black lines show medians, dark gray bands show inner 50^th^ percentiles, and light gray bands show inner 90^th^ percentiles.

Long-read sequencing yielded a median of 1.5E6 reads per sample (range 0.5–4.4 reads) (**Supplemental Table 2**). The median fragment length was 799 base pairs (maximum 5,043 base pairs), and the median average base quality per read was 14 (**Figures 2A, B**). This corresponded to a median of approximately 600 reads per allele for class I loci and 140 reads per allele for class II loci, reflecting the predominance of T lymphocytes, which express class I HLA genes but not class II, in peripheral blood (**Figure 2C**). 90%–100% of class I alleles and 73%–100% of class II alleles achieved ≥25X coverage at ≥90% of reference nucleotides (**Table 1**; **Supplemental Table 3**). Notably, we observed significant coverage bias (i.e., a relative excess) towards the 3’ end of all HLA loci, which has been previously reported with nanopore cDNA sequencing and may be related to the use of oligo(dT) primers for cDNA synthesis as well as read truncation during sequencing (**Figure 3**) (13–15).

**Table 1:** HLA gene coverage. Aggregated summary detailing the number of alleles analyzed per gene along with the proportion of alleles with 90% of target nucleotides achieving ≥10X, ≥25X, ≥50X, ≥75X, or ≥100X coverage.

Locus		Total Alleles	Distinct Alleles	Percent of Alleles with ≥90% of Nucleotides with ≥X Coverage				
				10X	25X	50X	75X	100X
Class I	*HLA-A*	30	7	100	100	90	87	77
	*HLA-B*	30	15	100	100	97	83	73
	*HLA-C*	30	11	97	90	77	70	63
Class II	*HLA-DPA1*	28	3	100	100	82	75	64
	*HLA-DPB1*	30	7	100	100	93	87	70
	*HLA-DQA1*	30	9	97	73	43	17	3
	*HLA-DQB1*	30	10	100	87	57	40	37
	*HLA-DRB1*	30	12	100	100	90	73	67
	*HLA-DRB3/4/5*	21	9	100	90	62	52	43

**Figure 4:**
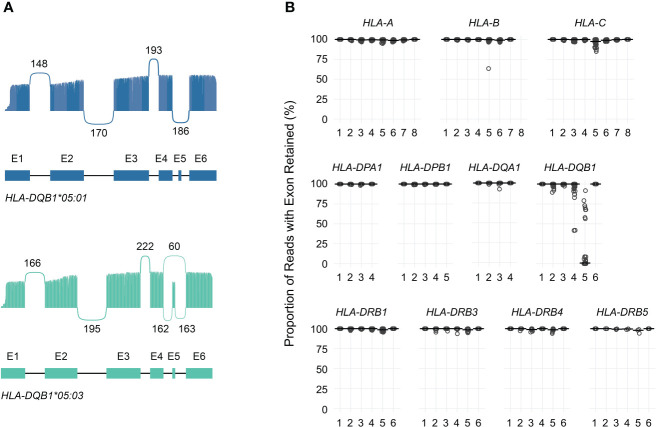
Quantification of HLA transcript isoforms. **(A)** Sashimi plots showing allele-specific exon utilization for *HLA-DQB1* for an example patient. Values indicate the number of times the indicated junctions were observed. **(B)** Dot plot showing, for each exon (x-axis), the proportion of reads in which that exon was retained (y-axis) for each gene analyzed. Points correspond to individual alleles (two per study subject). Horizontal bars correspond to medians.

**Figure 4A** shows allele-specific spliced alignments for *HLA-DQB1* in an individual sample (cDNA001) with a HLA-DQB1*05:03/05:01 genotype as an example. All reads aligning to either HLA-DQB1*05:01 or HLA-DQB1*05:03 included previously annotated exons 1 through 4 as well as exon 6. In contrast, approximately 0% of reads aligning to HLA-DQB1*05:01 and 73% of reads aligning to HLA-DQB1*05:03 included exon 5, consistent with allele-specific expression of transcript isoforms. Across the entire study population, loss of known exons in class I HLA genes was rare, with one sample showing loss of exon 5 in 37% of HLA-B*51:01 reads, two samples showing loss of exon 5 in 10%–13% of HLA-C*04:01 reads, and one sample showing loss of exon 5 in 15% of HLA-C*15:02 reads (**Supplemental Table 4**; **Figure 4B**).

**Figure 5:**
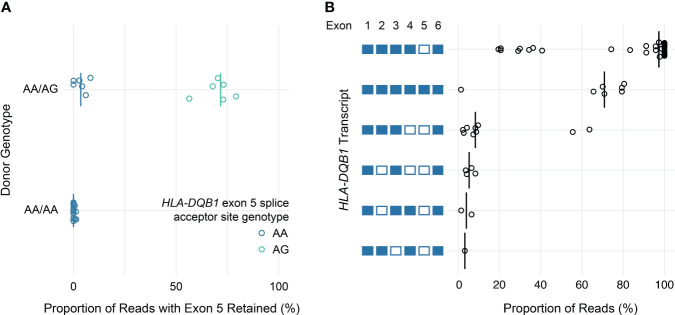
*HLA-DQB1* exon usage and full-length transcript quantification. **(A)** Dot plot showing estimated allele-specific exon 5 retention (x-axis) for *HLA-DQB1* alleles, grouped by donor genotype. Each point corresponds to an individual allele, colored by rs28688207 genotype (AG: splice acceptor intact; AA: splice acceptor lost). Vertical bars indicate medians. **(B)** Dot plot showing *HLA-DQB1* transcript utilization for full-length transcripts detected in the study cohort. Transcripts are represented by the schematic (left) where included exons are filled and excluded exons are open.

Similarly, loss of known exons among class II HLA genes was uncommon, with the exception of *HLA-DQB1*. Previous work has shown that the expression of exon 5 of *HLA-DQB1* is allele-specific and depends on a single nucleotide polymorphism in the fourth intron (rs28688207), which corresponds to a G to A substitution immediately 5’ of exon 5 that eliminates the splice acceptor site (16, 17). Partitioning the estimated expression of exon 5 in *HLA-DQB1* transcripts by rs28688207 genotype showed that this exon was selectively retained among donors with the G allele (i.e., alleles with an intact splice site) (**Figure 5A**). Specifically, this exon was retained in 72% of transcripts with the G allele among donors who were heterozygous for rs28688207, 4% of transcripts with the A allele among heterozygous donors, and 0% of donors who were homozygous for the A allele. Therefore, quantifying allele-specific *HLA-DQB1* transcripts with long-read RNA-seq recapitulates known variation in *HLA-DQB1* expression, suggesting that this may be a valid approach for characterizing HLA allele-specific transcript isoforms. As a caveat, the observation that 4% of reads aligning to the A allele show retention of exon 5 among heterozygous donors, but 0% of reads among homozygous donors, suggests modest cross-contamination in allele-specific expression estimates due to misaligned reads.

In addition to using nanopore sequencing to quantify allele-specific exon usage, we also specifically evaluated its ability to detect and quantify the expression of full-length transcripts. To illustrate this, **Figure 5B** shows the proportion of reads supporting distinct *HLA-DQB1* transcripts detected by at least one read in at least one sample. These data are generally consistent with the exon utilization data presented above. The majority of *HLA*-*DQB1* transcripts omit exon 5, while a subset of reads from donors with HLA-DQB1*05:03 and *06:01* alleles retain all six exons. *HLA*-*DQB1* transcripts that exclude exon 4 (seen in donors with HLA-DQB1*03:02, *03:03*, *06:02*, and *06:03*) were seen exclusively among transcripts that also exclude exon 5. All other *HLA*-*DQB1* transcripts were seen in only one or two reads in three or fewer donors, consistent with either low levels of expression or technical artifacts. Results for transcripts detected at all HLA loci are presented in **Supplemental Table 5**.

**Figure 6:**
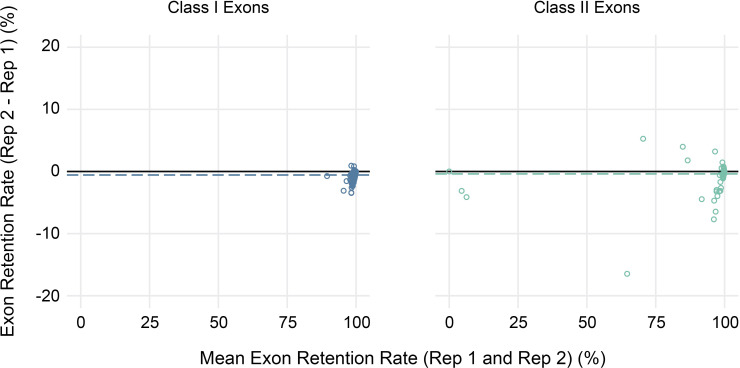
Reproducibility of exon usage estimates between replicates. Bland-Altman plot showing the mean coverage (x-axis) for all alleles across three sets of replicates (2 per subject) for class I (left) and class II (right) HLA genes versus the observed difference in estimated exon retention (y-axis).

Finally, to evaluate the reproducibility of estimated exon retention rates, replicate peripheral blood samples collected from three study participants at a second time point were also sequenced. Overall, replicates were highly concordant, with average absolute differences in estimated exon retention rates <1% for both class I and class II loci (range 0%–4% for class I and 0%–17% for class II) (**Figure 6**). Furthermore, 95% of exon retention rate estimates were within 2% between replicates for class I exons and 4% for class II exons.

## Discussion

While short-read RNA or cDNA sequencing methods can robustly quantify gene expression, they cannot accurately profile most transcript isoforms according to benchmark studies due to large variation in expression levels and the sequencing depth required for analysis (18–20). Surveying the splicing landscape of HLA alleles is further hindered by allelic diversity and heterogeneity in expression (8). In this cross-sectional study, we analyzed full-length cDNA sequencing data from Oxford Nanopore Technologies’ MinION to characterize alternative splicing of HLA transcripts at the allele level. Despite the high error rate of MinION sequencing reads and significant variation in the coverage of alleles in this study, we show that allele-specific HLA splice isoforms can be efficiently detected and quantified. We reported exon utilization across 81 common HLA alleles from 11 class I and II loci, quantified the relative abundance of phased transcript isoforms, and correlated the splicing of exon 5 of the *HLA-DQB1* gene with known allelic sequence variation. These data highlight the potential of nanopore cDNA sequencing to efficiently profile the differential splice isoforms from highly polymorphic HLA genes.

Nanopore long-read sequencing has been applied to examine alternative splice isoforms in murine and human tissues, but few studies have examined HLA transcript isoforms in detail. Sessegolo et al. studied the transcriptomes of mouse brain and liver using both short-read and nanopore sequencing of RNA and cDNA and found that nanopore sequencing was more efficient at quantifying full-length transcripts and more strongly correlated with spike-in controls (13). Byrne and colleagues studied the splice isoforms for B cell surface receptors, such as CD19, CD20, CD37, and IGH, in murine B1a cells (21). After benchmarking their approach using synthetic transcripts, these authors showed that nanopore 2D reads (now deprecated) could identify and quantify complex isoforms at the single-cell level, while Illumina reads failed to form complete contigs or produced contigs not detected by full-length nanopore reads. Both of these studies highlight the relative strengths of nanopore RNA-seq in capturing the diversity of splice isoforms with biological relevance. In addition, nanopore RNA and/or cDNA sequencing was recently used to characterize alternative splicing of *PKD1* transcripts in human kidney tissues and *CACNA1C* gene transcripts in human brain tissue (22, 23).

In our study, we focused on splice isoforms of HLA genes across a group of diverse alleles in human lymphocytes. With a straightforward workflow and bioinformatic pipeline, we consistently detected the loss of exon 5 in transcripts from *HLA-DQB1* alleles with a G>A substitution at the splice acceptor site preceding exon 5, confirming the impact of the AA genotype on mRNA splicing (24). Interestingly, even for alleles with an intact splice acceptor site such as HLA-DQB1*05:03, exon 5 was excluded in over 25% of transcripts. Exon 5 of *HLA-DQB1* and *HLA-DRB1* genes are equally small (only 24 base pairs), and exon 5 is completely retained in all *HLA-DRB1* alleles tested in our study. These data suggest that the omission of exon 5 in a subset of *HLA-DQB1* transcripts with the AG genotype is not due to technical artifacts related to the small size of exon 5. It is possible that additional *cis-*acting elements in *HLA-DQB1* alleles with the AG genotype may suppress splicing and cause occasional skipping of exon 5 in the final transcripts. Regarding the physiologic relevance of the alternative splicing of exon 5 in *HLA-DQB1* alleles, this particular exon encodes the middle portion of cytoplasmic tails of HLA-DQ beta chains, which include a motif predicted to mediate cyclic AMP signaling (25). However, the impact of exon 5 exclusion on HLA-DQ expression levels, antigen presentation, and immune-related disorders remains elusive and warrants further study.

Exon 4 of *HLA-DQB1*, which encodes the transmembrane domain of the HLA-DQ beta chain, has been reported to be skipped in transcripts in cell lines carrying HLA-DQB1*03, 04, 06 allele groups but not HLA-DQB1*02 or *05* (26). The corresponding proteins lacking the transmembrane domain were predicted to be secreted and were subsequently found in supernatants of cultured cell lines (16). This phenomenon was shown to be regulated by *cis*-acting elements in intron 3, specifically the branchpoint sequence haplotypes, polypyrimidine tract variants, and guanosine repeats upstream of the branchpoint (26, 27). In the current study, we observed low rates of *HLA-DQB1* exon 4 exclusion (2%–8%) specifically in donors with HLA-DQB1*03 or *06* but not HLA-DQB1*02 or *05*, consistent with previous work. These data support alternative splicing as a source of soluble DQ proteins, limited to certain alleles and representing the minor form compared to the transmembrane DQ proteins in peripheral blood lymphocytes.

Splice isoforms of *HLA-A* and *-C* have also been reported previously. Transcripts without exon 5, which encodes the transmembrane domain of class I HLA, were detected in various tumor cells and normal cells, encoding soluble HLA-A23 and A24 antigens (28). Subsequent studies have repeatedly confirmed HLA-A23 and A24 as high-secretor HLA molecules in Caucasians (29). Ehlers et al. recently reported HLA-C*04 and C*16 splice isoforms lacking exon 5, which was identified in cDNA extracted from human peripheral blood (30). The abundance of these isoforms, predicted to encode soluble HLA-C antigens, is not known. Although alternative splicing can produce soluble HLA molecules, there are other mechanisms that may play a larger role, such as shedding from the cell membrane or enzymatic cleavage of the extracellular domains (29, 31). In our study, we observed low levels of exon 5 skipping in samples with HLA-A*23 (3%, n = 1) or A*24 (2%–6%, n = 3). Similarly, low levels of exon 5 skipping were seen in samples with HLA-C*01 (0%–3%, n = 3), C*03 (1%–6%, n = 5), C*04 (11%–15%, n = 4), C*05 (0%–2%, n = 3), C*06 (3%, n = 1), C*07 (0%–4%, n = 8), C*08 (0%–1%, n = 2), and C*15 (3%–13%, n = 3). Finally, we also noted low levels of exon 5 exclusion from samples with B*51 (4%–9%, n = 2). Taken together, these data suggest that exon 5 skipping in class I HLA transcripts occurs in an allele-specific manner at low to undetectable levels in human peripheral blood lymphocytes and is unlikely to be a major source of soluble class I antigens.

This proof-of-concept study has several limitations. The sample size is small and covers a limited set of HLA alleles. The cDNA libraries were prepared using bulk lymphocytes enriched from peripheral blood samples, which does not provide lineage-specific or single-cell information. Although long reads were generated to cover target genes at a median depth of 140 (class II) to 600 (class I) reads, we observed varying degrees of 3’ bias across all HLA genes examined. This bias may be related to the use of oligo(dT) primers for cDNA synthesis combined with reduced efficiency of reverse transcription across the GC-rich exons 2 and 3 of class I genes and exon 2 of class II genes. The resulting reduction in 5’ coverage may limit the sensitivity of our approach to estimating exon utilization in this region. In addition, by requiring exons to be flanked by retained exons to be scored as excluded, our analysis of exon utilization is restricted to internal exons. Furthermore, while full cDNA reference sequences were available for all alleles in this study, some HLA alleles have incomplete reference sequences (e.g., limited to exons 2 and 3), and inferred full-length sequences need to be used with the approach we describe. Similarly, our method is limited to the analysis of known HLA alleles. Finally, allele-specific splice isoforms due to alternative polyadenylation in the 3’-UTR were not detected (32). In the future, many of these limitations could be addressed by identifying and quantifying transcripts *de novo* (i.e., without relying on reference sequences), but additional method development and validation in this area is needed.

In summary, this study demonstrates the feasibility of using nanopore sequencing to detect and quantify allele-specific HLA transcript isoforms in primary human lymphocytes. In the cohort analyzed, we find that the expression of alternative HLA transcripts is generally rare with the notable exception of *HLA-DQB1*, which demonstrates allele-specific exclusion of exon 5 (consistent with previous reports). Going forward, it will be important to apply methods like this to larger cohorts to characterize HLA transcript isoform usage across a broader set of HLA alleles and diverse genetic backgrounds. In addition, this method can be used to analyze other tissues and/or purified cell populations to identify tissue- and cell-type-specific expression of HLA transcripts. Similarly, it can be applied to specimens from both healthy and diseased individuals to determine if perturbations in the HLA transcript repertoire are associated with specific pathological states. Ultimately, this method may have clinical utility in providing more detailed information about expressed HLA antigens, which could inform more accurate assessments of histocompatibility in the setting of organ transplantation.

## Data availability statement

The RNA-seq data analyzed in this study (raw reads) are publicly available via the National Center for Biotechnology Information (NCBI) Sequence Read Archive (SRA) under accession PRJNA1004569.

## Author contributions

AH, MM, CL, and EW designed the study. MM performed the experiments, and AH analyzed the data. AH, CL, and EW prepared the manuscript. AH, MM, CL, and EW reviewed and edited the manuscript.
