# Supplementary material for: Allele-specific quantification of human leukocyte antigen transcript isoforms by nanopore sequencing
Source: Front Immunol. 2023 Aug 18;14:1199618. doi: 10.3389/fimmu.2023.1199618 (PMC10471969; doi:10.3389/fimmu.2023.1199618)
Supplement: Supplementary file 1 [file DataSheet_1.pdf]

Supplementary Material

Allele-Specific Quantification of Human Leukocyte Antigen Transcript Isoforms by Nanopore Sequencing

Andrew E.O. Hughes[1], Maureen C. Montgomery[2], Chang Liu[1] and Eric T. Weimer[2-3]

[1]Department of Pathology and Immunology, Washington University School of Medicine, St. Louis, MO
[2]Molecular Immunology Laboratory, McLendon Clinical Laboratories, University of North Carolina Hospitals, Chapel Hill, NC
[3]Department of Pathology & Laboratory Medicine, University of North Carolina at Chapel Hill School of Medicine, Chapel Hill, NC

*** Correspondence:**

Eric T. Weimer
University of North Carolina Hospitals
101 Manning Drive, Room 1053, East Wing
Chapel Hill, NC 27514
Ph: 984-747-5773
E-mail: eric_weimer@med.unc.edu

Chang Liu
Washington University School of Medicine
660 S. Euclid Ave, Box 8118
St. Louis, MO 63110
Ph: 314-747-5773
Email: cliu32@wustl.edu

Supplemental Tables

Supplemental Table 1: Sample HLA Types. Reference HLA types determined for all samples in the study. NT: not typed.

Supplemental Table 2: Number of HLA Reads Per Allele. Coverage per allele for all samples in the study.

Supplemental Table 3: Individual-Level Allele Representation. Proportion of target loci (i.e., the proportion of nucleotides in each reference sequence) covered at ≥10, ≥25, ≥50, ≥75, and ≥100X for each allele and sample in the study.

Supplemental Table 4: Individual-Level Exon Utilization. Proportion of reads with retained exons for each allele and sample in the study (based on canonical exons).

Supplemental Table 5: Individual-Level Transcript Census. Number of reads supporting the presence of specific transcripts for each allele and sample in the study. Retained exons are indicated by E1, E2, E3, etc. and skipped exons are indicated by underscores (_).

Supplemental Table 1: Sample HLA Types

Sample	*HLA-A*	*HLA-B*	*HLA-C*	*HLA-DPA1*	*HLA-DPB1*	*HLA-DQA1*	*HLA-DQB1*	*HLA-DRB1*	*HLA-DRB3/4/5*
cDNA001	A*02:01:01<br>A*11:01:01	B*35:01:01<br>B*40:01:02	C*03:04:01<br>C*04:01:01	DPA1*01:03:01<br>DPA1*01:03:01	DPB1*02:01P<br>DPB1*03:01P	DQA1*01:01:01<br>DQA1*01:04:01	DQB1*05:01:01<br>DQB1*05:03:01	DRB1*01:01:01<br>DRB1*14:54:01	DRB3*02:02:01
cDNA002	A*02:01:01<br>A*11:01:01	B*27:05:02<br>B*55:01:01	C*01:02:01<br>C*03:03:01	DPA1*01:03:01<br>DPA1*01:03:01	DPB1*02:01P<br>DPB1*03:01P	DQA1*01:01:01<br>DQA1*01:04:01	DQB1*05:01:01<br>DQB1*05:03:01	DRB1*01:01:01<br>DRB1*14:54:01	DRB3*02:02:01
cDNA003	A*02:01:01<br>A*23:01:01	B*35:01:01<br>B*49:01:01	C*04:01:01<br>C*07:01:01	DPA1*01:03:01<br>DPA1*01:03:01	DPB1*04:01:01<br>DPB1*04:01:01	DQA1*01:04:01<br>DQA1*02:01:01	DQB1*02:02:01<br>DQB1*05:03:01	DRB1*07:01:01<br>DRB1*14:01:01	DRB3*02:24<br>DRB4*01:01
cDNA005	A*02:01:01<br>A*03:01:01	B*07:02:01<br>B*15:01:01	C*01:02:01<br>C*07:01:01	DPA1*01:03:01<br>DPA1*01:03:01	DPB1*02:01P<br>DPB1*04:02P	DQA1*03:03:01<br>DQA1*02:01:01	DQB1*03:02:01<br>DQB1*02:02:01	DRB1*04:01:01<br>DRB1*07:01:01	DRB4*01:01:01<br>DRB4*01:03:01
cDNA006	A*01:01:01<br>A*02:01:01	B*07:02:01<br>B*08:01:01	C*07:01:01<br>C*07:02:01	DPA1*01:03:01<br>DPA1*02:06	DPB1*03:01:01<br>DPB1*05:01:01	DQA1*01:02:01<br>DQA1*05:01:01	DQB1*02:01:01<br>DQB1*06:02:01	DRB1*03:01:01<br>DRB1*15:01:01	DRB3*01:01:02<br>DRB5*01:01:01
cDNA008	A*02:01:01<br>A*24:02:01	B*07:02:01<br>B*57:01:01	C*06:02:01<br>C*07:02:01	DPA1*01:03:01<br>DPA1*01:03:01	DPB1*04:01P<br>DPB1*04:02P	DQA1*01:01:01<br>DQA1*02:01:01	DQB1*03:03:02<br>DQB1*05:01:01	DRB1*01:01:01<br>DRB1*07:01:01	DRB4*01:03:01N
cDNA009	A*02:01:01<br>A*03:01:01	B*08:01:01<br>B*35:01:01	C*04:01:01<br>C*07:01:01	DPA1*01:03:01<br>DPA1*02:01:01	DPB1*03:01:01<br>DPB1*09:01:01	DQA1*01:01:01<br>DQA1*05:01:01	DQB1*02:01:01<br>DQB1*05:01:01	DRB1*01:01:01<br>DRB1*03:01:01	DRB3*01:01:02
cDNA010	A*03:01<br>A*11:01	B*44:02<br>B*51:01	C*05:01<br>C*15:02	NT	DPB1*03:01<br>DPB1*14:01	DQA1*01:03<br>DQA1*03:01	DQB1*03:02<br>DQB1*06:03	DRB1*04:04<br>DRB1*13:01	NT
cDNA011	A*02:01:01<br>A*24:02:01	B*44:29<br>B*51:01:01	C*05:01:01<br>C*15:02:01	DPA1*01:03:01<br>DPA1*01:03:01	DPB1*04:01:01<br>DPB1*04:01:01	DQA1*02:01:01<br>DQA1*03:01:01	DQB1*02:02:01<br>DQB1*03:02:01	DRB1*04:01:01<br>DRB1*07:01:01	DRB4*01:01:01<br>DRB4*01:03:01
cDNA012	A*01:01:01<br>A*02:01:01	B*14:02:01<br>B*15:17:01	C*07:01:02<br>C*08:02:01	DPA1*01:03:01<br>DPA1*02:06	DPB1*02:01:02<br>DPB1*05:01:01	DQA1*01:01:02<br>DQA1*01:02:01	DQB1*05:01:01<br>DQB1*06:04:01	DRB1*01:02:01<br>DRB1*13:02:01	DRB3*03:01:01
cDNA013	A*02:01:01<br>A*30:04:01	B*14:01:01<br>B*40:01:02	C*03:04:01<br>C*08:02:01	DPA1*01:03:01<br>DPA1*01:03:01	DPB1*04:01:01<br>DPB1*04:01:01	DQA1*01:02:01<br>DQA1*01:03:01	DQB1*06:01:01<br>DQB1*06:02:01	DRB1*15:01:01<br>DRB1*15:02P	DRB5*01:01:01<br>DRB5*01:02
cDNA014	A*03:01:01<br>A*03:01:01	B*07:02:01<br>B*07:02:01	C*07:02:01<br>C*07:02:01	DPA1*01:03:01<br>DPA1*01:03:01	DPB1*04:01:01<br>DPB1*04:01:01	DQA1*01:02:01<br>DQA1*01:02:01	DQB1*06:02:01<br>DQB1*06:02:01	DRB1*15:01:01<br>DRB1*15:01:01	DRB5*01:01:01<br>DRB5*01:01:01

Supplemental Table 2: Number of HLA Reads Per Allele

Sample	Total Coverage	*HLA-A*	*HLA-B*	*HLA-C*	*HLA-DPA1*	*HLA-DPB1*	*HLA-DQA1*	*HLA-DQB1*	*HLA-DRB1*	*HLA-DRB3/4/5*
cDNA001	2,627,191	1,311	1,643	1,452	212	418	75	222	569	188
		914	1,873	1,708	212	475	61	214	195	
cDNA002	3,714,866	1,149	1,305	845	297	632	77	232	575	167
		962	1,648	1,154	297	554	88	227	214	
cDNA003	3,031,735	1,282	1,454	1,375	260	364	87	286	616	156
		1,294	1,336	608	260	364	196	259	262	309
cDNA005	969,316	521	358	227	198	202	88	101	183	112
		391	227	209	198	175	108	84	213	89
cDNA006	1,455,648	583	775	455	239	353	64	83	559	222
		691	936	463	149	316	42	294	755	782
cDNA008	3,761,507	340	389	715	62	181	28	57	245	96
		410	476	239	62	129	135	138	319	
cDNA009	4,386,208	1,367	1,573	1,459	337	848	103	187	814	175
		974	1,598	875	282	792	118	322	717	
cDNA010	791,564	200	318	485		111	18	34	107	
		189	221	661		120	37	38	84	
cDNA011	669,100	299	431	490	50	81	52	35	148	70
		355	328	617	50	81	69	38	152	74
cDNA012	828,177	279	465	191	87	122	22	32	138	43
		422	323	844	41	105	21	55	93	
cDNA013	1,779,238	1,103	818	601	356	368	63	99	260	235
		817	1,109	1,746	356	368	77	135	252	244
cDNA014	510,733	178	164	66	62	58	16	34	43	44
		178	164	66	62	58	16	34	43	44
